# Exploring the total flavones of *Abelmoschus manihot* against IAV-induced lung inflammation by network pharmacology

**DOI:** 10.1186/s12906-022-03509-0

**Published:** 2022-02-05

**Authors:** Yanan Gao, Zihao Liang, Nianyin Lv, Jinjun Shan, Huihui Zhou, Junfeng Zhang, Liyun Shi

**Affiliations:** 1 School of Medicine, Nanjing University of Chinese Medicine, Nanjing, 210046 China; 2 The First School of Clinical Medicine, The Affiliated Hospital of Nanjing University of Chinese Medicine, Nanjing, 210029 China; 3 School of Medicine, Zhejiang Shuren University, Hangzhou, 310015 Zhejiang China

**Keywords:** Network pharmacology, IAV, TFA, Anti-inflammation, Anti-virus

## Abstract

**Background:**

*Abelmoschus manihot (L.) Medicus* (AM) is a medicinal plant with various biological activities, including anti-inflammatory, antioxidant, antiviral and immunomodulatory. Previous studies have identified total flavones as the primary bioactive ingredient of AM (termed TFA). However, its role and mechanism in counteracting Influenza A virus (IAV) infection are yet to be explored. Therefore, the study aims to study the antiviral and anti-inflammatory effects of TFA on IAV in vitro and in vivo.

**Methods:**

A network pharmacology-based approach was applied to identify the antiviral mechanism of TFA against IAV. For the mechanism validation, the cytopathic effect reduction assay evaluated the antiviral activity of TFA in vitro. Meanwhile, the mice were intranasally infected with IAV to induce lung infection. The antiviral effect of TFA was observed in vivo. Further investigation whether the reprogramming microbiome in the TFA treatment group affected antiviral, we conducted a microbial-transfer study with co-housing experiments.

**Results:**

By applying the network pharmacology-based methods (PPI, GO, and KEGG), we identified 167 potential targets of TFA action, among which 62 targets were related to IAV pathogenesis. A core network containing the pro-inflammatory TNFα, IL-6, IL-1β, MAPKs, and RIG-I receptor signaling pathway was further confirmed as the crucial targets for anti-influenza efficacy of TFA. We demonstrate that TFA provided profound protection against pulmonary IAV infection, which alleviated inflammatory responses, decreased MAPK signaling pathway and expedited viral eradiation.

**Conclusions:**

Our study unveils a pivotal role for TFA in controlling viral infection and dampening pathology, making it a promising strategy for treating IAV-induced pneumonia.

**Supplementary Information:**

The online version contains supplementary material available at 10.1186/s12906-022-03509-0.

## Background

Influenza A virus (IAV) causes an infectious respiratory disease in humans, resulting in an annual worldwide epidemic of high morbidity and mortality [1, 2]. IAV primary infection and replication in the respiratory tract epithelial cells [3]. The lung macrophage phagocytosis cellular debris and activated to fight off the virus [4, 5]. The macrophage and epithelial cells in the lung generate a variety of pro-inflammatory cytokines, for instance, interleukin-1β (IL-1β), tumor necrosis factor α (TNF-α) and interleukin-6 (IL-6), and so on [6]. This release causes more neutrophils and macrophages to be attracted and activated into the lung tissue, priming the innate immune response critical to removing and clearing viral particles [7, 8]. Pathogenesis of influenza viruses induced acute lung injury, including direct damage from virus replication or indirect damage resulting from the influenza-induced cytokine storm [9, 10].

In recent years, the exploitation of anti-flu medicine has increasingly focused on host immune regulation, especially anti-inflammatory. Traditional Chinese medicine (TCM) affords underlying candidates for exploiting such drugs because they suppress virus replication and reduce inflammation [11–14]. The total flavones of *Abelmoschus manihot* (TFA), as the significant composition of *Abelmoschus manihot* (AM), are extensively applied to deal with anti-inflammatory, anti-viral, anti-bacterial, and anti-tumor [15]. In the past two decades, the HuangKui capsule extracted from AM has been approved by the China State Food and Drug Administration (Z19990040) to treat chronic kidney disease [16]. In clinical trials, TFA plays a vital role in effectively improving nephrotic syndrome, renal inflammation, membranous nephropathy, and purpuric nephritis [17, 18]. However, it is unclear whether TFA could play against IAV and its mechanisms remain unknown.

Recently, network pharmacology has been widely used to research TCM. A set of TCM network pharmacology methods was established to sequence disease-related genes, predict compounds’ targeted distribution and pharmacological action, reveal the common modularized association of drug target-pathway-disease, and virtually screen synergistic compounds of TCM preparations [19, 20]. Considering the components of TFA alone, it is necessary to clarify the anti-influenza mechanism of TFA utilizing network pharmacology.

It has been reported that the main bioactive constituents of TFA include isoquercitrin, myricetin, quercetin, and so on [21]. Since flavonoids are strongly hydrophilic, they usually have low absorption and low bioavailability [22]. Flavonoids are absorbed in the small intestine [23]. Recent evidence suggests that intestinal microbes may further transform flavonoids into metabolites with increased or decreased biological activity [24]. Quercetin and hesperidin have been found to prevent obesity and associated metabolic diseases by increasing probiotics, reducing pathogenic bacteria, and regulating the intestinal microbiota composition [25, 26]. Desaminotyrosine (DAT), a metabolite associated with microorganisms, is a degradation product of quercetin that protects the body from Influenza by enhancing Type I interferon signaling, weakening pulmonary immunopathology [27]. The complex interaction of intestinal flora with flavonoids may be necessary for the pharmacological activity of these natural products.

Therefore, this study aims to elucidate the underlying mechanism of TFA in IAV treatment using a network pharmacological approach. We also carried out in vitro and in vivo experiments to verify these predictions. Our findings prove for the first time that TFA can improve pulmonary inflammation caused by IAV. Furthermore, the intestinal microbes may convert TFA into specific metabolites to produce part of the anti-influenza effect.

## Materials and methods

### Reagent and antibodies

TFA was extracted from flowers of *A. manihot* by the Department of Chinese Materia Medica, Nanjing University of Chinese Medicine (Nanjing, China). The TFA extraction process is as follows: three extractions with 70% alcohol for 50 min each, and the supernatant after centrifugation was evaporated and freeze-dried to obtain a powder [28]. The quality of TFA was evaluated with a fingerprint analysis by high performance liquid chromatography (HPLC), and the method was performed as previously described [29]. The TFA powder was first dissolved in dimethyl sulfoxide (DMSO) and then diluted with PBS before use. Methyl Thiazolyl Tetrazolium (MTT), Desaminotyrosine (DAT) was purchased from Sigma (St.Louis, USA).

Anti-CD11b, anti-CD11c, anti-F4/80, and anti-Ly6G were purchased from Invitrogen (Carlsbad, CA, USA). Antibodies against JNK (#9252), p-JNK (T183/Y185, #4668), p38 (#9212), p-p38 (T180/Y182, #9211), Erk1/2 (#4695), p-Erk1/2 (Thr202/Tyr204, #4370), RIG-I (#3743), MAVS (#24930), p-TBK1 (#5483), TBK1 (#38066), p-IRF3 (#37829), IRF3 (#4302), and β-actin (#3700) were purchased from Cell Signaling Technology (Danvers, MA).

### Cell and virus

MDCK (Madin-Darby canine kidney) and MH-S (the mouse alveolar macrophage) cells were cultivated in DMEM or RPMI-1640 containing 10% fetal bovine serum, penicillin of 100 U/mL, and streptomycin of 10 μg/mL (Gibco, Carlsbad, CA, USA) at 37 °C and 5% CO_2_. Influenza A virus strain PR8 (A/Puerto Rico/8/34, H1N1), a gift by Professor Sun Ren (Zhejiang University School of Medicine, China), was amplified in MDCK cells and titers were tested by 50% tissue culture infectious dose (TCID_50_). In addition, infections were carried in serum-free DMEM containing the antibiotics and 2 μg/ml of TPCK-treated trypsin (Gibco, Carlsbad, CA, USA).

### Cytotoxicity assay

The cytotoxicity of TFA on MDCK cells was determined using an MTT assay. The cells (2 × 10^5^ per well) were seeded in a 96-well plate at 37 °C and 5% CO_2_. Replaced with a fresh medium containing continuous diluted TFA and incubated the cells at 37 °C for 48 h. Add 20 μL of a 5 mg/ml MTT solution to each well, and incubate the cells for a further 4 h at 37 °C. Subsequently, the supernatant was aspirated, and formazan crystals were solubilized with DMSO (100 μL/well). The absorbance of OD value was measured using a microplate reader at 490 nm (Bio-Rad Laboratories, California, USA). The 50% toxic concentration (TC_50_) was determined using GraphPad Prism 8 software.

### Antiviral assay in vitro

The cytopathic effect reduction assay evaluated the antiviral activity of TFA. MDCK cells (2 × 10^5^ per well) were seeded in a 96-well plate. Cells infected with 100 TCID_50_ of the virus at 37 °C for 2 h. The supernatant was aspirated, and MDCK cells were cultured with 100 μl of serum-free MEM, including 2 μg/ml of TPCK-treated trypsin and serially diluted TFA at 37 °C. At 48 h post-infection, the half-maximal inhibitory concentration (IC_50_) values were calculated by the MTT assay.

In some experiments, MDCK or MH-S cells were cultured in 24-well plates and infected with the virus (MOI = 0.1) for 2 h; then, TFA or DAT was added to the medium, respectively. Finally, the cells were harvested at 8 h post-infection.

### In vivo treatment of mice

Specific pathogen-free (SPF) female C57BL/6 mice 6 to 8 weeks old were used in this study. The mice were gavage TFA (125, 250, 500 mg/kg) or PBS daily for 7 days (*n* = 6). Two days later, the virus was diluted in sterile PBS and administered intranasally in a volume of 100 μl per mouse to anesthetized mice maintained in an upright position. PR8 was given at a dose of 5000 TCID_50_ per 100 μl, which caused ∼10% weight loss and no mortality. The infected mice were euthanized 3 days post-infection.

### Bronchoalveolar lavage fluid (BALF) harvest and cell preparation

The trachea of mice was exposed and cannulated with a 24-gauge plastic catheter. BALF was obtained by washing the lung 3 times with 1 ml aliquots of saline through a tracheal cannula. BALF was collected, and then cells were separated and counted. The protein concentration of BALF supernatants was determined by bicinchoninic acid assay following the manufacturer’s instructions.

### Flow cytometry analysis

Harvest BALF and prepare single-cell suspension (1 × 10^6^ cells/mL) in the staining buffer. Add fluorescent-conjugated antibody (CD11b and F4/80 for macrophage; CD11b and Ly6G for neutrophil; CD11c for dendritic cells) and incubate on ice for 30 min. Unconjugated antibodies were removed by washing the cells with staining buffer. The cells were resuspended in 500 μL staining buffer and determined by flow analysis.

### Lung histopathology

Approximately 5 mm × 5 mm lung tissue was fixed in 4% (w/v) formalin and embedded in paraffin. Lung tissues were cut into slices, stained with hematoxylin and eosin (H&E), and examined microscopically.

### Real-time PCR

Total RNA was extracted from mouse lung tissues or cell lysates using Trizol reagent (Invitrogen). cDNA was generated using an HiScript II OneStep RT-PCR Kit (Vazyme, Nanjing, China). SYBR Green PCR Master Mix (YEASEN, Shanghai, China) was applied to quantitate the mRNA levels. The primers for analyses are listed in Table 1, and β-actin is the internal control. The reactions were performed in triplicate, and relative mRNA expression was calculated using the 2^−△△Ct^ method.

**Table 1:** Sequences of Quantitative PCR primers

Gene	Primer (5′ → 3′)
*β-actin*1)	F: CTCATGAAGATCCTGACCGAG
	R: AGTCTAGAGCAACATAGCACAG
*IL-1β*1)	F: GAAATGCCACCTTTTGACAGTG
	R: TGGATGCTCTCATCAGGACAG
*IL-6*	F: CCACTTCACAAGTCGGAGGCTTA
	R: AGTGCATCATCGTTGTTCATAC
*TNF-α*	F: AAGGCCGGGGTGTCCTGGAG
	R: AGGCCAGGTGGGGACAGCTC
*IFN-β*	F: CAGCTCCAAGAAAGGACGAAC
	R: GGCAGTGTAACTCTTCTGCAT
*Mx2*	F: GAGGCTCTTCAGAATGAGCAAA
	R: CTCTGCGGTCAGTCTCTCT
*Osa1*	F: CGCACTGGTACCAACTGTGT
	R: CTCCCATACTCCCAGGCATA
*Osa2*1)	F: TTGAAGAGGAATACATGCGGAAG
	R: GGGTCTGCATTACTGGCACTT

### Western blot analysis

Lung tissues or MH-S Cells were lysed in RIPA lysis buffer supplemented with protease and phosphatase inhibitors to extract protein. Equal amounts of protein lysate in the samples were separated by SDS-PAGE (Bio-Rad), transferred onto polyvinylidene difluoride membranes (PVDF, pore size 0.45 μM; Bio-Rad). After blocking in Tris-buffered saline with Tween-20 (TBST) containing 5% bovine serum albumin (BSA). Incubate the membranes with the primary antibody and the secondary antibody conjugated with horseradish peroxidase (CST). The signals were visualized with an ECL western blotting kit (YEASEN).

### Co-housing experiment

6 to 8 weeks old mice were randomly divided into five groups (*n* = 6). Mice were administrated with tap water (Ctrl group), PBS, or TFA for 1 week. PBS treated mice and TFA treated mice were either co-housed (Co-housing) or housed singly (Single-housing) for 1 week before IAV infection. The virus infection method is as described above.

### Fecal DAT analysis

Stool samples were extracted at 100 mg/ml tissue in 80% methanol, then centrifuged for clarification and filtered. Concentrations of DAT in stool samples collected from mice were determined by mass spectrometry [27].

### Network pharmacological analysis of TFA

The six main flavonoids (isoquercetin, quercetin, quercetin-3′-O-glucoside, myricetin, rutin, and hyperoside) in TFA were distinguished via HPLC and identified their chemical structures [21, 30, 31]. The relevant target proteins were acquired from Traditional Chinese Medicine Systems Pharmacology (TCMSP) (https://tcmsp-e.com) [32]. We mapped the proteins correlated with gene symbols, conforming to the Uniprot database (https://www.uniprot.org). A set of genes associated with influenza was chosen based on OMIM (https://www.omim.org), GeneCards (https://www.genecards.org), and DisGeNET (https://www.disgenet.org) databases. Intersections were made between TFA target genes and influenza-related genes and visualized with Venn diagrams. We employed the Search Tool for the Retrieval of Interacting Genes (STRING) database (https://string-db.org) to execute a protein-protein interaction (PPI) network. The results were visualized by Cytoscape software (https://cytoscape.org). We conducted Gene Ontology (GO) [33, 34] and the Kyoto Encyclopedia of Genes and Genomes (KEGG) [35–37] pathway enrichment analysis to understand the function and potential pathways.

### Statistics

The values were calculated as the mean ± standard deviation (SD) in 3 independent experiments. Data were analyzed using GraphPad Prism 8 and SPSS 22 Software. Comparison of data from two groups was analyzed by analysis of two-tailed Student’s *t*-test. Differences were considered statistically significant when *p* < 0.05.

## Results

### Identification of the crucial molecules targeted by TFA to control IAV

**Fig. 1:**
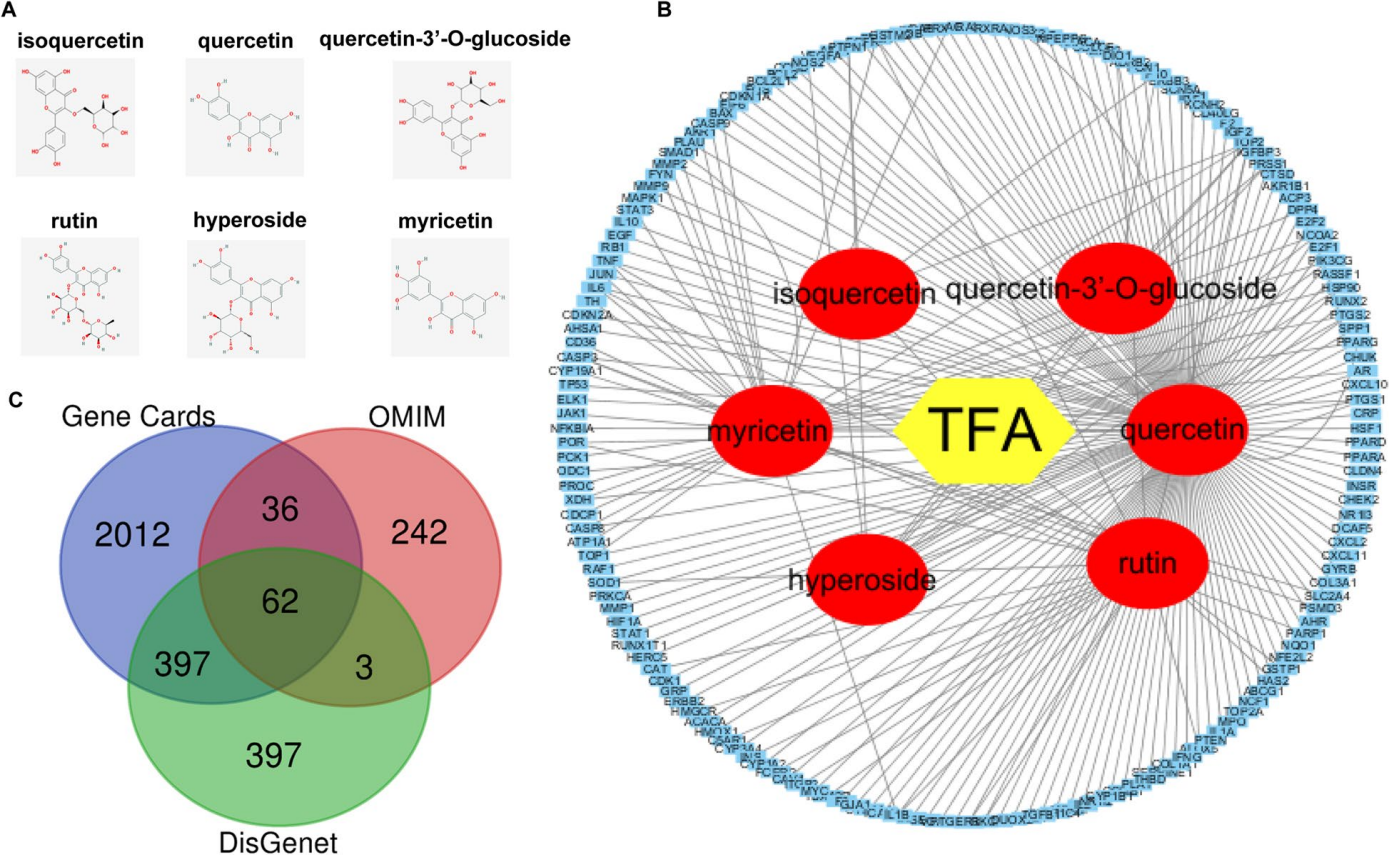
Screening of anti-influenza components in TFA. **A** 2-D structures of the six main flavonoids in TFA. **B** 167 candidate targets of TFA were identified to establish the components-target interaction network. The red nodes stand for the six main flavonoids in TFA, and the blue nodes represent the protein targets on which the component acts. **C** 62 overlapping genes related to Influenza were identified from GeneCards, OMIM, and DisGeNET

Given that TFA exerts pharmacological effects through multiple targets, it is necessary to explore the targets accurately. HPLC fingerprint of TFA was shown the six different peaks (isoquercetin, quercetin, quercetin-3′-O-glucoside, myricetin, rutin, and hyperoside) (SFig.1). The chemical structures of six main flavonoids in TFA were identified in TCMSP (Fig. 1A). A compound-target interaction network was established by Cytoscape 3.8 to reveal the correlation between TFA and target genes. One hundred sixty-seven nodes in this network indicate that TFA may exert its treatment effect through multiple compounds and varieties of target genes (Fig. 1B). 2507, 343, or 859 genes related to influenza were respectively identified from GeneCards, OMIM, and DisGeNET databases in total (Fig. 1C). The results of the Draw Venn diagram suggested that 62 overlapping targets were screened from these databases.

### PPI network analysis reveals the anti-inflammatory effect of TFA

**Fig. 2:**
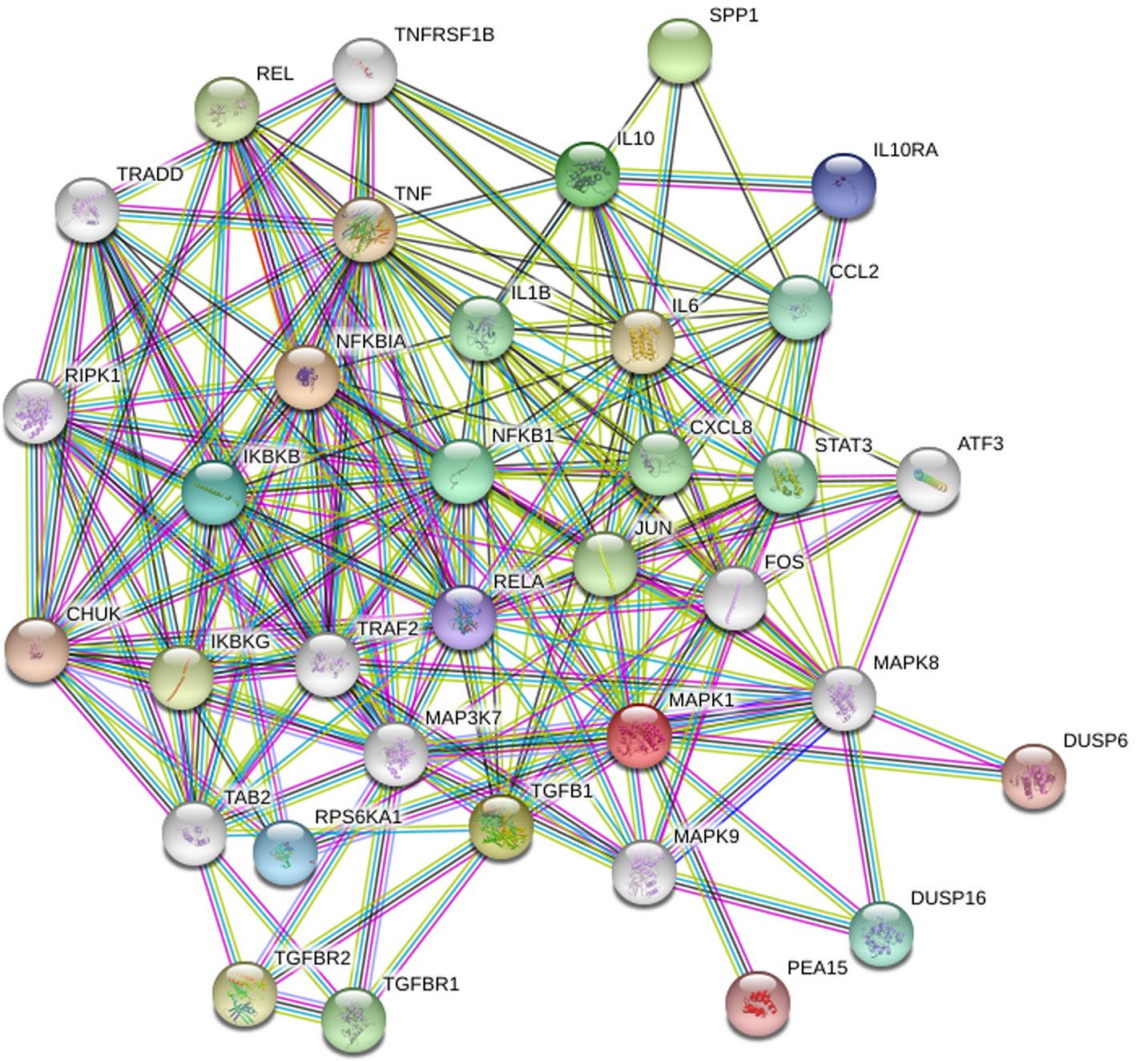
Identification of TFA targets using the protein-protein interaction network. The mapping of PPI network was generated by STRING. Network nodes represented proteins. Color nodes represented query protein and the first shell, the white nodes represented the second shell, and the filled nodes represented some known or predicted 3D structure. The edges represented protein-protein binding

The PPI network reflects the relationship between molecules in the cell. It provides valuable information for studying molecular mechanisms in physiological and pathological states. Based on this, we imported common gene targets of TFA and IAV into the STRING database. Use qualified *Homo sapiens* with a confidence of 0.7 and hidden discontinuous nodes in the network to perform protein interaction network analysis and download the updated TSV format. This file has been imported into Cytoscape for topology attribute analysis. There were 35 nodes and 206 edges in the PPI network and then visualized by Cytoscape (Fig. 2). The analysis using CytoHubba, TNF, IL10, IL-6, IL-1B, JUN, and MAPK1 was found to have the highest average scores and identified as crucial targets for the anti-influenza efficacy of TFA.

### KEGG and GO analysis supports the combined anti-viral and anti-inflammatory effects of TFA

**Fig. 3:**
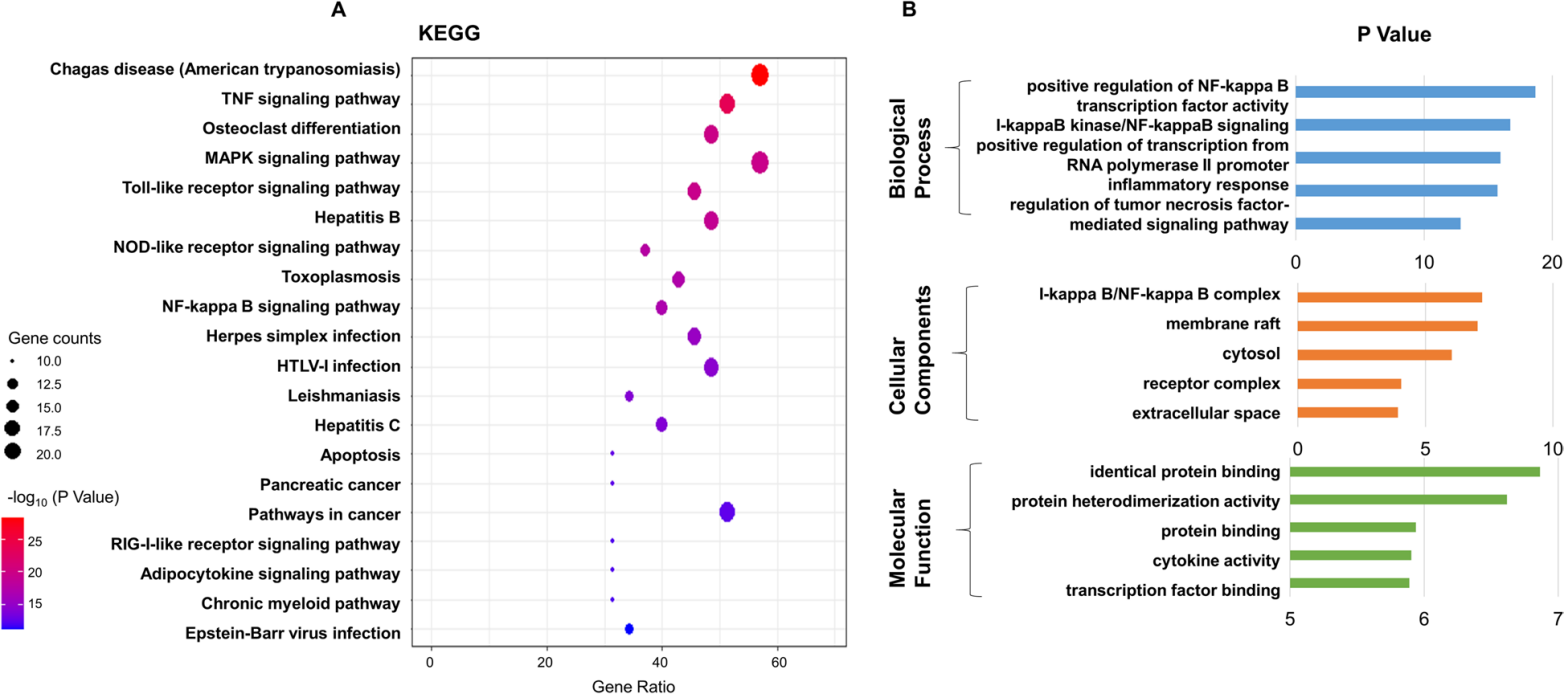
KEGG and GO analysis for targets of TFA against IAV. **A** There was a bubble chart of the first 20 KEGG pathways. The horizontal axis of the bubble chart represented the rate of the critical targets involved to the total number of targets in the pathway. Bubble size represented the number of core targets involved in the pathway, and the color from blue to red indicated that the *P* value was from small to large. **B** The top 5 terms for biological processes, cell components, and molecular function with *P* < 0.05 are shown

To clarify the overall role of TFA in infection, we selected 35 candidate targets for KEGG path enrichment analysis. KEGG pathway analysis revealed that 35 targets of TFA against Influenza were mainly enriched in 73 signaling pathways (*P* < 0.05). Moreover, 20 signaling pathways visualized by Ominshare were directly involved. They might be the critical mechanism of TFA against Influenza, including TNF signaling pathway, MAPK signaling pathway, NF-Kappa B signaling pathway, Toll-like receptor signaling pathway, RIG-I-like receptor signaling pathway, and so on (Fig. 3A). GO annotation is carried out from three aspects, including biological process (BP), cell composition (CC), and molecular function (MF). The enrichment results included 166 BP items, 22 CC items, 42 MF items and showed the first five items with significantly adjusted *P* values (Fig. 3B). Central BP included the positive regulation of NF-kappa B transcription factor activity, inflammatory response, regulation of tumor necrosis factor-mediated signaling pathway, etc. Main CC involved I-kappa B/NF-kappa B complex, membrane raft, cytosol, receptor complex, and other cell components. Main MF covered identical protein binding, protein heterodimerization activity, cytokine activity, etc.

### Anti-inflammatory and antiviral activity of TFA in vitro

**Fig. 4:**
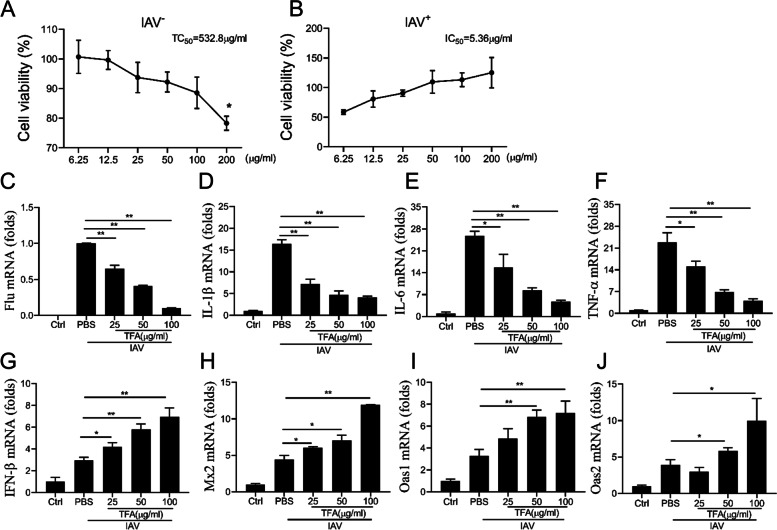
TFA displays anti-inflammatory and anti-viral effects in vitro. **A** The cytotoxicity of TFA on MDCK cells was tested by MTT assay. **B** The cytopathic effect inhibition assay evaluated the antiviral activity of TFA by MTT test. MDCK cells were cultivated in a 48-well plate and infected with PR8 (MOI = 0.01) for 2 h, then TFA was added to the medium. The cells were harvested at 8 h post-infection. The mRNA levels of (**C**) Viral, (**D**) IL-1β, (**E**) IL-6, (**F**) TNF-α, (**G**) IFN-β, (**H**) Mx2, (**I**) Oas1, and (**J**) Oas2 were determined by real-time PCR. β-actin was used as a control. The value are shown as means ± SD of three individual experiments. *P < 0.05, **P < 0.01

The cell viability of MDCK cells after TFA was measured by MTT assay. TFA showed unapparent cytotoxicity at concentrations up to 100 μg/ml (Fig. 4A). To investigate the antiviral effect of TFA on influenza virus PR8, we infected MDCK cells with 100 TCID_50_ of the virus and incubated them with various concentrations of TFA or 48 h. TFA inhibited the replication of IAV with an IC_50_ value of 5.36 μg/ml (Fig. 4B). MDCK cells were infected with PR8 and treated with TFA (25, 50, 100 μg/ml) for observed anti-inflammatory effects of TFA. The results showed that TFA reduced viral RNA and the mRNA levels of IL-1β, IL-6, and TNF-α in a dose-dependent manner (Fig. 4C-F). To further evaluate the activation of antiviral response genes, we tested the mRNA expression of IFN-β, Mx2, Oas1, and Oas2. It was shown that TFA significantly increased the mRNA levels of these genes (Fig. 4G-J).

### TFA suppressed IAV-induced lung inflammation in mice

**Fig. 5:**
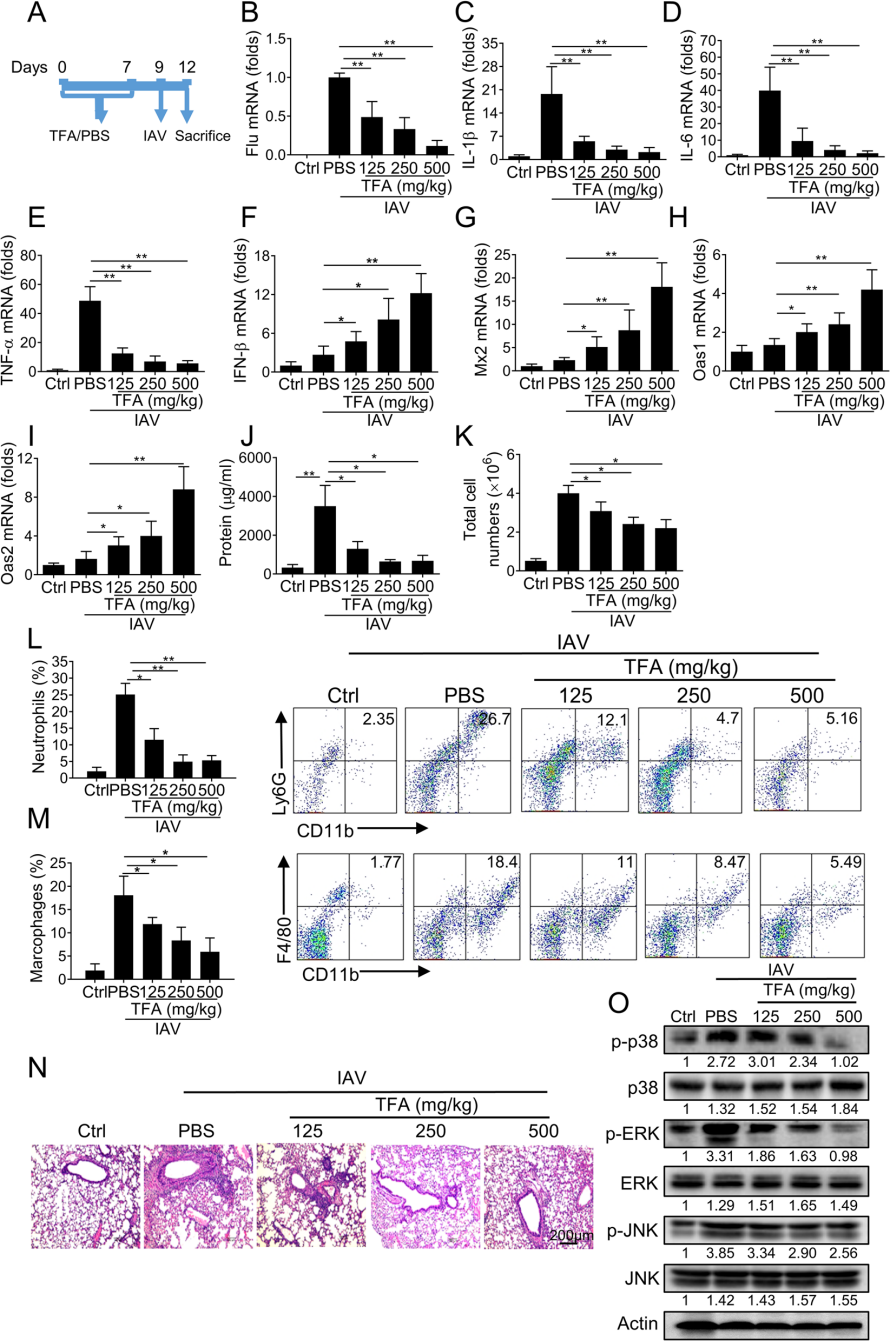
TFA treatment remarkably alleviates IAV-induced lung inflammation in mice. TFA (125, 250, 500 mg/kg) or PBS was gavage daily for 7 days, and PR8 was intranasally administrated at a dose of 5000 TCID_50_ per mouse (*n* = 6 mice per group). **A** The experimental scheme. **B** The viral mRNA, the pro-inflammatory cytokines (**C**) IL-1β, (**D**) IL-6, and (**E**) TNF-α and the type I IFN signaling related genes (**F**) IFN-β, (**G**) Mx2, (**H**) Oas1, and (**I**) Oas2 were determined by real-time PCR. β-actin was used as a control. **J** The protein and (**K**) the total cells in BALF were determined. The proportions of (**L**) CD11b^+^Ly6G^+^ neutrophils and (M) CD11b^+^F4/80^+^ macrophages in BALF were detected by flow cytometry. **N** The pathological changes were evaluated by H&E staining. **O** Phosphorylated and total levels of p38, ERK1/2, and JNK were observed in lung tissue by western blot. The intensities of bands relative to the control were measured with ImageJ software, and the results are shown below. The value are shown as means ± SD of three individual experiments. *P < 0.05, **P < 0.01

We have studied its latent therapeutic value in IAV infection to determine whether TFA has an antiviral effect. Mice were treated PBS or TFA (125, 250, 500 mg/kg) for 7 days in advance and then infected with IAV. Compared with the PBS group, mice in the TFA treatment group significantly reduced viral RNA on day 3 post-infection (Fig. 5A-B). To observe IAV-induced lung inflammation, we evaluated the levels of pro-inflammatory cytokines. The results manifest that the mRNA levels of pro-inflammatory cytokines (IL-1β, IL-6, and TNF-α) in the PBS group increased significantly after infection. However, TFA greatly reduced the increase of these cytokines (Fig. 5C-E). To assessment the type I IFN signaling, we detected the mRNA levels of IFN-β, Mx2, Oas1, and Oas2. Administration of TFA could elevate Type I IFN signaling in the lung in a dose-dependent manner compared to the PBS group (Fig. 5F-I). Mice in the TFA group decreased the protein and total cells compared to the PBS group (Fig. 5J, K). Subsequently, we measured the proportion of neutrophils, macrophages, or dendritic cells (DCs) in BALF. On day 3 post-infection, we observed extensive infiltration of neutrophils, macrophages, or DCs compared with the uninfected controls, reflecting airway inflammation. TFA treatment caused significant changes in neutrophils or macrophages rations (Fig. 5L, M). However, there was no difference in the proportions of DCs between TFA treatment groups (Supplement Fig. 2). Uncontrolled pulmonary inflammation exacerbates the pathology caused by IAV infection. We further assess the severity of lung inflammation by histopathological analysis. TFA administration can significantly alleviate the pathological changes (Fig. 5N). Meanwhile, we analyzed the activity of MAPKs, which are the main effectors that mediate pro-inflammatory signals. TFA also significantly reduced the phosphorylation of p38, ERK1/2, and JNK, the key MAPKs for transcription and production of pro-inflammatory mediators (Fig. 5O). In summary, our results revealed that TFA significantly protected mice with IAV-induced pneumonia.

### Co-housing with TFA-treated mice confers the protection for IAV infection

**Fig. 6:**
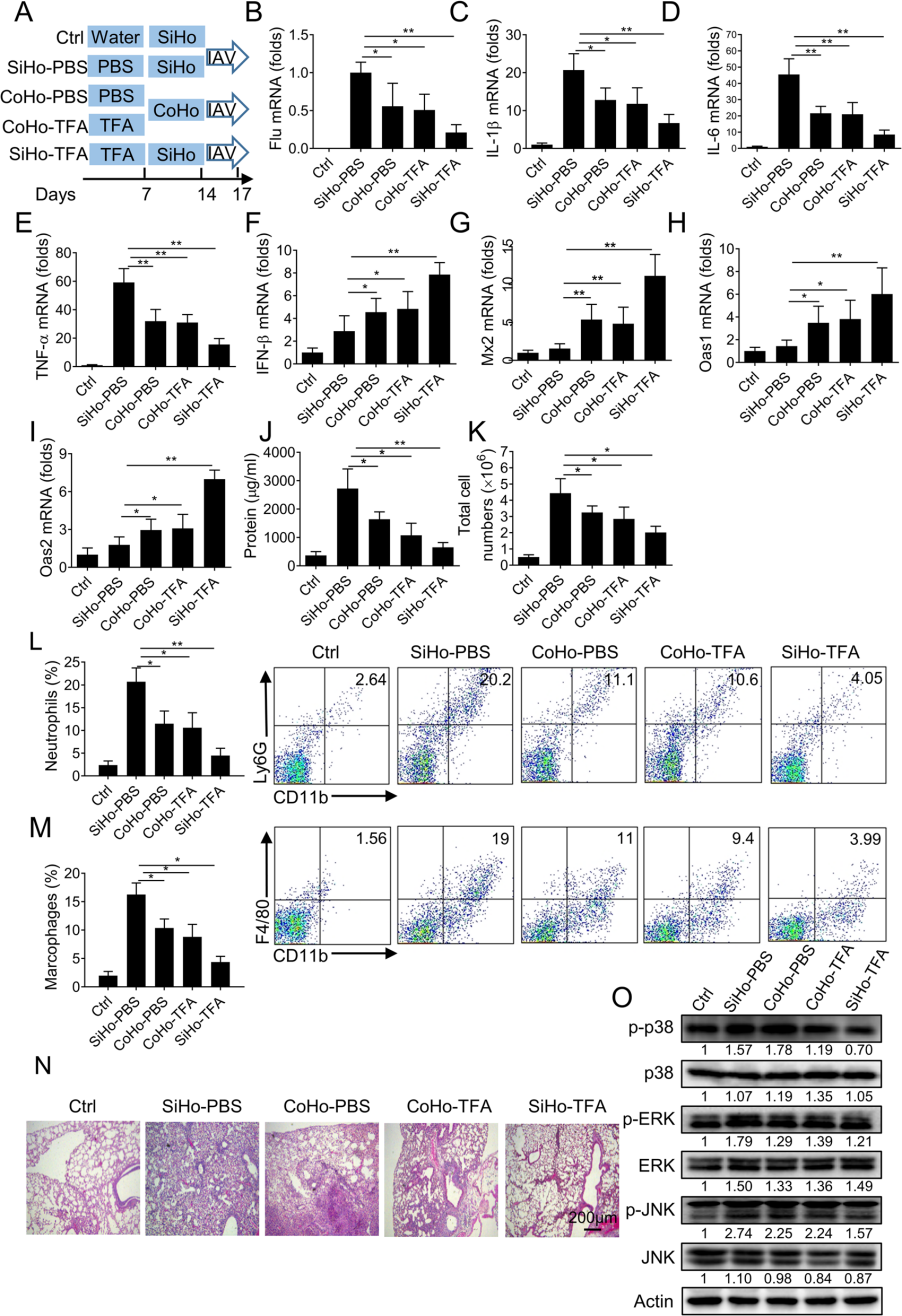
Co-housing with TFA-treated mice confers the protection of IAV-induced lung inflammation. **A** Diagram of the co-housing experiment. Randomly divided the mice into five groups and administrated with water (Ctrl group), PBS, or TFA (250 mg/kg) for 1 week, separately. Before infected with IAV, TFA-treated mice or PBS-treated mice were reared alone (Single-housing) or co-housed (Co-housing) for 1 week (*n* = 6 mice per group). **B** The viral mRNA was examined. The mRNA expression levels of (**C**) IL-1β, (**D**) IL-6, (**E**) TNF-α, (**F**) IFN-β, (**G**) Mx2, (**H**) Oas1, and (**I**) Oas2 were assessed by real-time PCR. β-actin was used as a control. **J** The protein and **K** the total cells in BALF were determined. The proportions of (**L**) CD11b^+^Ly6G^+^ neutrophils and (**M**) CD11b^+^F4/80^+^ macrophages in BALF were detected by flow cytometry. **N** The pathological changes were evaluated by H&E staining. **O** Phosphorylated and total levels of p38, ERK1/2, and JNK measured in lung tissue by western blot. The intensities of bands relative to the control were measured with ImageJ software, and the results are shown below. The value are shown as means ± SD of three individual trials. *P < 0.05, **P < 0.01

To further explore whether the gut microbiome in the TFA treatment group affected the antiviral, we conducted a microbial-transfer study with co-housing mice, leading to microbiome exchange via coprophagia. Raise mice pretreated with TFA for 1 week alone or together with mice treated with PBS for 1 week, and then carry out IAV infection (Fig. 6A). The mice in the SiHo-TFA group were pretreated with TFA, a microbial inducer of antiviral effect in intestinal tissue. In co-housing experiments, the CoHo-TFA group were donor mice, and the CoHo-PBS group were recipient mice. As expected, co-housing of PBS and TFA treated mice (CoHo-PBS and CoHo-TFA) can protect against IAV infection compared with the single housing group (SiHo-PBS). Mice in the CoHo-PBS group significantly decreased viral RNA compared with the SiHo-PBS group (Fig. 6B). Mice in the CoHo-PBS group significantly reduced the mRNA expression levels of pro-inflammatory cytokines (IL-1β, IL-6, and TNF-α) compared with the SiHo-PBS group after infection (Fig. 6C-E). In the meantime, mice in the CoHo-PBS group significantly increased the mRNA levels of IFN-β, Mx2, Oas1, and Oas2 compared with the SiHo-PBS group (Fig. 6F-I). We observed that the protein, total cells, and the number of neutrophils or macrophages in the CoHo-PBS group were significantly less than in the SiHo-PBS group (Fig. 6J-M). Mice in the CoHo-PBS group ameliorated the pathological changes compared with the SiHo-PBS group (Fig. 6N). Mice in the CoHo-PBS group also significantly reduced the phosphorylation of p38, ERK1/2, and JNK compared with the SiHo-PBS group (Fig. 6O). The results indicate that the antiviral effects of TFA could be influenced by co-housing via a microbiome-dependent mechanism affecting TFA.

### The metabolite DAT of TFA exhibits anti-inflammatory and antiviral effects in vitro

**Fig. 7:**
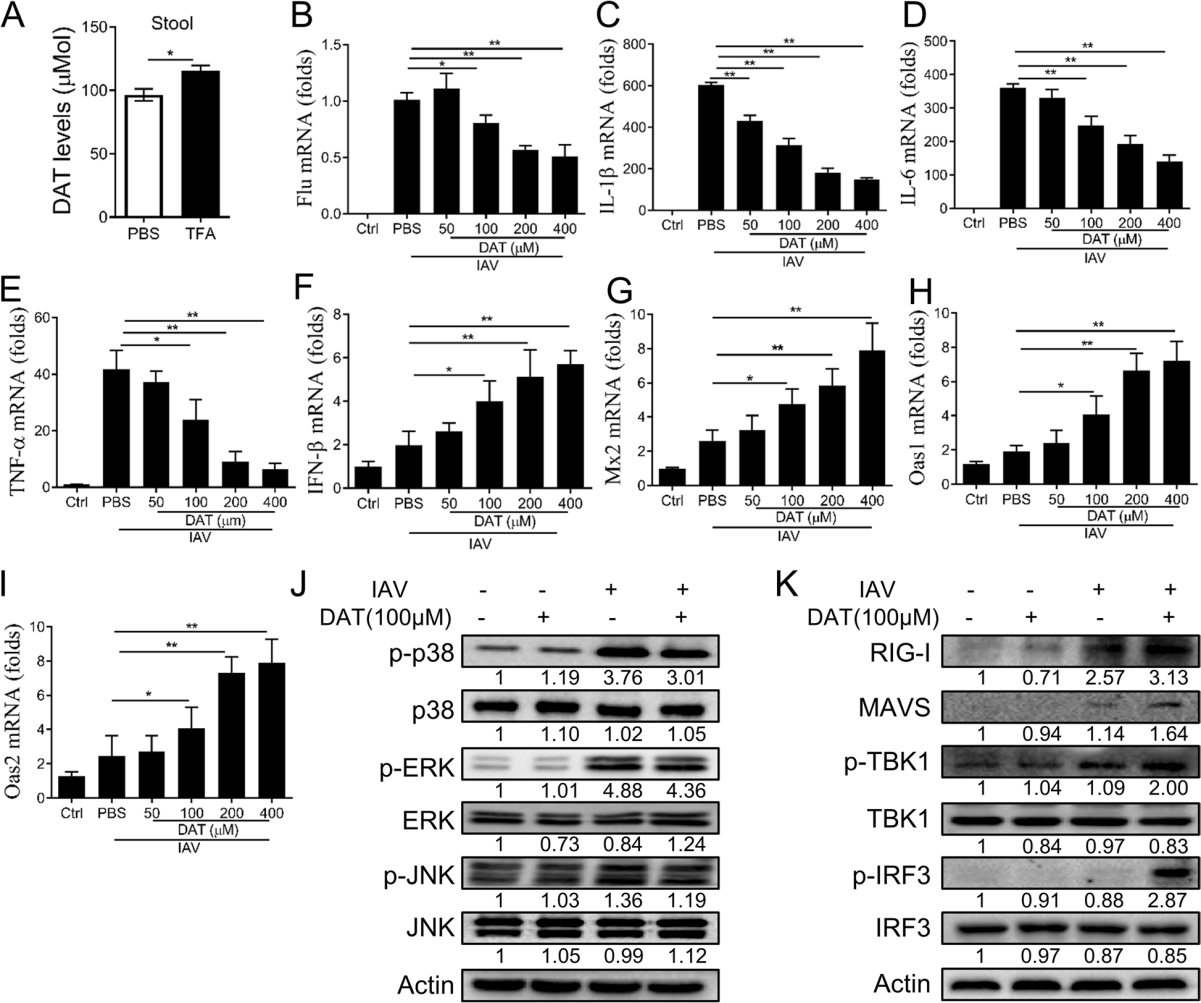
DAT displays anti-inflammatory and antiviral effects in vitro. **A** The fecal DAT levels of mice treated with vehicle (PBS) or TFA for 1 week were measured by mass spectroscopy (*n* = 3 mice per group). MH-S cells were untreated (PBS) or treated with DAT and then infected with PR8 (MOI = 0.01). The mRNA levels of (**B**) viral, (**C**) IL-1β, (**D**) IL-6, (**E**) TNF-α, (**F**) IFN-β, (**G**) Mx2, (**H**) Oas1, and (**I**) Oas2 were determined by real-time PCR. β-actin was used as a control. **J** Phosphorylated and total levels of p38, ERK1/2, and JNK were measured in MH-S cells by western blot. **K** Immunoblotting of RIG-I, MAVS, total and phosphorylated TBK1, and IRF3 measured in MH-S cells. The intensities of bands relative to the control were measured with ImageJ software, and the results are shown below. The value are shown as means ± SD of three individual trials. *P < 0.05, **P < 0.01

Mice were given 250 mg/kg TFA a week in advance, and DAT levels in their feces were measured. We observed that the TFA group’s DAT was higher than that of the Ctrl group in feces (Fig. 7A). Alveolar macrophage (AM) plays a key role in regulating host defense, pulmonary inflammation, and tissue damage following respiratory virus infection [38]. We examined the effects of DAT on the anti-inflammatory and anti-viral responses triggered by IAV exposure. To assess the anti-inflammatory and anti-viral effects of DAT on PR8 virus, MH-S cells were infected with PR8 influenza virus and treated with DAT (50, 100, 200, 400 μM). The results showed that DAT reduced viral RNA and the mRNA levels of IL-1β, IL-6, and TNF-α in dose-dependent (Fig. 7B-E). We assessed the expression of IFN-inducible antiviral response genes, and the results showed that DAT significantly increased the mRNA levels of IFN-β, Mx2, Oas1, and Oas2 compared with PBS (Fig. 7F-I). We analyzed the activity of MAPKs and the retinoic acid-inducible gene-I (RIG-1)-like receptor RNA-sensing pathway by immunoblot in MH-S cells infected with IAV alone or in combination with DAT. DAT decreased the activation of phosphorylation of p38, ERK1/2, and JNK and increased the activation of RIG-I, MAVS, p-TBK1, and p-IRF3 compared with IAV infection alone (Fig. 7J, K).

## Discussion

Network pharmacology is a powerful tool for identifying the different components of Chinese medicine and investigating the mechanism of Chinese medicine [39, 40]. Previous studies have shown that TFA has a wide range of pharmacological effects, including anti-inflammatory, analgesic, antioxidant, liver protection, kidney protection, and cardiovascular protection [17, 30, 41–44]. However, TFA has not been systematically studied to verify its anti-influenza activity. The main bioactive components of TFA detected by HPLC include isoquercetin, quercetin, quercetin 3′-O-glucoside, rutin, myricetin, and hyperoside, which are consistent with previous studies [44]. We obtained 167 potential TFA targets and 62 influenza-related targets from databases. With the merger of PPI network, we have identified a core network, including TNF, IL-6, IL-1B, JUN, and MAPK1, as crucial targets of the anti-influenza efficacy of TFA. The data uncovered several critical signaling pathways through GO and KEGG analysis, such as TNF signaling pathway, MAPK signaling pathway, NF-Kappa B signaling pathway, Toll-like receptor signaling pathway, and RIG-I-like receptor signaling pathway.

However, network-based pharmacological analysis requires more evidence to assess the potential anti-influenza effects of TFA. Subsequently, the antiviral effects of TFA were measured in virus-infected cells. TFA was inoculated 2 h prior to IAV infection with MDCK cells, and a cytopathic effect inhibition assay was determined. The results showed that TFA strongly inhibited virus adherence to cells and reduced the pro-inflammatory cytokines when administered before viral infection in vitro. We further used a mouse model of IAV infection in vivo. It was found that TFA decreased viral load and the mRNA levels of pro-inflammatory cytokines (IL-1β, IL-6, and TNF-α) and inhibited MAPK signaling pathway. These findings suggest that TFA may exert an anti-influenza effect by suppressing lung inflammation. Therefore, our findings indicated that network pharmacology analysis and experiments complement each other to illustrate the mechanism of TFA in the treatment of IAV.

Influenza A virus induces high pro-inflammatory cytokines and chemokines that cause extensive infiltration and lung tissue damage [45–47]. In the early stage of influenza virus infection, neutrophils, macrophages, and DCs have been shown to clear influenza virus via phagocytosis or promotion of adaptive responses. When encountering pathogens, macrophages produce pro-inflammatory cytokines to recruit neutrophils into the alveolar spaces. Although neutrophils are essential for clearing pathogens, excessive neutrophil accumulation also correlated with disease severity following IAV infection. The high numbers of recruited neutrophils may enrich inflammation and cause massive tissue/organ damage. We found that TFA markedly decreased the total cells, the proportion of neutrophils and macrophages, and protein in BALF. Several reports have shown that effective anti-influence strategies reduce viral load and inhibit respiratory damage caused by inflammation [48–50]. Traditional Chinese medicine may be a potential for drug design and for finding alternative sources. For example, Lianhuaqingwen Capsule (LH-C) has proven effective against influenza and reduced the pro-inflammatory cytokines (IL-6 and TNF-α) in the lungs of mice [51, 52]. Fufang-Yinhua-Jiedu Granules (FFYH) was used to treat influenza, and the antiviral effect may be attributed to the suppression of inflammatory cytokine expression by regulating the TLR7/MyD88/NF-κB signaling pathway [53].

TFA is administered orally, and we hypothesized that TFA transformed by gut microbes into specific metabolites prevent influenza pathogenesis. Consistent with intended results, we further found that TFA reduced IAV-induced inflammation by targeting the intestinal flora when mice shared symbiotic bacteria through a co-housing experiment. Our previous research has indicated that the extracts of *Abelmoschus manihot* notably relieved colitis in mice induced by dextran sulfate sodium by regulating the intestinal flora’s composition and increasing the abundance and levels of the intestinal flora that produce straight-chain fatty acids (especially butyric acid and acetic acid). Besides, treatment with the extracts of *Abelmoschus manihot* notably reduced the mRNA levels of pro-inflammatory cytokines, for instance, IL-1β, IL-6, TNF-α, and IL-17 [54]. In chronic renal failure rats, TFA administration improved renal injury, remodeled gut microbiota dysbiosis, including regulated intestinal-derived metabolites such as D-serine, D-amino acid oxidase, L-serine, and serine racemase, suppressed the levels of pro-inflammation (TNF-α and IL-1β) [29]. The intestinal flora produces many small diffusible metabolites, which enter the systemic circulation to exert their effects further [51].

Since DAT is a quercetin degradation product by gut microbiota [27], we observed the anti-viral and anti-inflammatory effects of DAT in MH-S cells. RIG-I signaling plays a vital role in establishing an antiviral state that limits influenza virus replication [55, 56]. RIG-I detects the virus and triggers MAVS (mitochondrial antiviral signaling protein) activation of TBK1 (TANK-binding kinase 1). TBK1 phosphorylates interferon regulatory factor 3 (IRF3) to induce type I IFN (IFN-α/β) transcriptionally. In addition, IRF3 binding IFN-stimulated response elements (ISREs) to induce additional antiviral genes, such as Mx2, Oas1, and Oas2 [57, 58]. We found that DAT decreased the activation of phosphorylation of p38, ERK1/2, and JNK and increased the activation of RIG-I, MAVS, p-TBK1, and p-IRF3. Therefore, DAT inhibited inflammation via MAPK pathway and enhanced type I IFN signaling through activated RIG-I partly. As our research is still inadequate, further studies should be carried out to clarify the role of DAT in vivo.

In conclusion, we found that TFA can protect against the influenza virus predicted by network pharmacology. This research was the first to prove that TFA could inhibit IAV infection by inhibiting the release of inflammatory factors and suppressing MAPK signaling pathway. Furthermore, our results provide new insights that TFA may be transformed into specific metabolites by gut microbiota to improve lung inflammation. However, future research is required to explore the underlying mechanisms. These findings could help us fight IAV infections and provide a new drug therapy for the future.

## Conclusion

Based on the multi-component and multi-target action mode of traditional Chinese medicine, and according to the principle of network pharmacology, this study systematically explored the biologically active components and anti-inflammatory mechanisms antiviral effects of TFA. By applying methods based on network pharmacology, we identified 167 potential TFA targets, 62 of which are related to the pathogenesis of IAV. The core network, including the pro-inflammatory TNFα, IL-6, IL-1β, MAPK, and RIG-I receptor signaling pathways, is further confirmed as the critical target of TFA anti-influenza efficacy. We prove that TFA treatment provides profound protection against pulmonary IAV infection, reducing inflammation and accelerated virus clearance. Our research reveals the critical role of TFA in controlling viral infections and alleviating pathology, making it a promising strategy for treating IAV-induced pneumonia.

## Supplementary Information

**Additional file 1: Supplementary Figure 1.** HPLC fingerprint of total Total flavones of *Abelmoschus manihot (L.) medicus*. 1 Rutin, 2 hyperoside, 3 isoquercetin, 4 Myricetin, 5 quercetin-3′-O-glucoside, 6 quercetin. **Supplementary Figure 2.** The proportions of dentric cells in BALF. TFA (125, 250, 500 mg/kg) or PBS was gavage daily for 7 days, and PR8 was intranasally administrated at a dose of 5000 TCID50 per mouse (*n* = 3 mice per group). The proportions of CD11c + dentric cells in BALF were detected by flow cytometry. The value are shown as means ± SD of three individual experiments.

**Additional file 2: Supplementary Figure 3.** Gels/blots are cropped for the clear presentation of results. Samples derived from the same experiment and gels/blots were processed in parallel. The Uncropped Blot in Fig. 5O. The red arrow indicates the location of target bands. **Supplementary Figure 4.** Gels/blots are cropped for the clear presentation of results. Samples derived from the same experiment and gels/blots were processed in parallel. The Uncropped Blot in Fig. 6O. The red arrow indicates the location of target bands. **Supplementary Figure 5.** Gels/blots are cropped for the clear presentation of results. Samples derived from the same experiment and gels/blots were processed in parallel. The Uncropped Blot in Fig. 7J. The red arrow indicates the location of target bands. **Supplementary Figure 6.** Gels/blots are cropped for the clear presentation of results. Samples derived from the same experiment and gels/blots were processed in parallel. The Uncropped Blot in Fig. 7K. The red arrow indicates the location of target bands.

## Data Availability

The datasets used and/or analysed during the current study are available from the corresponding author on reasonable request.

## Abbreviations

IAV: Influenza A virus
AM: *Abelmoschus manihot*
DAT: Desaminotyrosine
IL-1β: Interleukin-1β
IL-6: Interleukin-6
TNF-α: Tumor necrosis factor α
TCM: Traditional Chinese medicine
TFA: The total flavone of *Abelmoschus manihot*
RIG-I: Retinoic acid-inducible gene-I
MAVS: Mitochondrial antiviral signaling protein
TBK1: TANK-binding kinase 1
IRF3: Interferon regulatory factor 3
ISREs: IFN-stimulated response elements
