# Supplementary material for: Exploring the total flavones of Abelmoschus manihot against IAV-induced lung inflammation by network pharmacology
Source: BMC Complement Med Ther. 2022 Feb 5;22:36. doi: 10.1186/s12906-022-03509-0 (PMC8817495; doi:10.1186/s12906-022-03509-0)
Supplement: Supplementary file 1 — Additional file 1: Supplementary Figure 1. HPLC fingerprint of total Total flavones of Abelmoschus manihot (L.) medicus. 1 Rutin, 2 hyperoside, 3 isoquercetin, 4 Myricetin, 5 quercetin-3′-O-glucoside, 6 quercetin. Supplementary Figure 2. The proportions of dentric cells in BALF. TFA (125, 250, 500 mg/kg) or PBS was gavage daily for 7 days, and PR8 was intranasally administrated at a dose of 5000 TCID50 per mouse (n = 3 mice per group). The proportions of CD11c + dentric cells in BALF were detected by flow cytometry. The value are shown as means ± SD of three individual experiments. [file 12906_2022_3509_MOESM1_ESM.docx]

**Supplementary Figure 1**

**
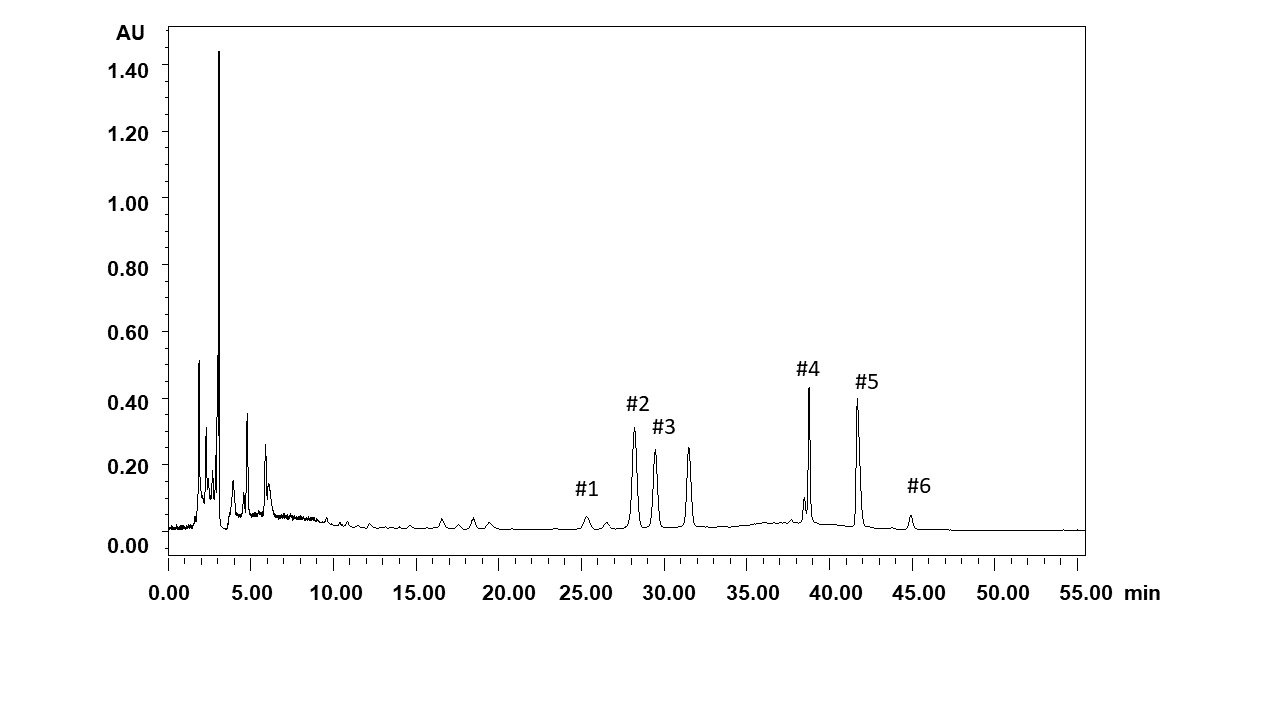
**

**HPLC fingerprint of total Total** **flavones of *Abelmoschus manihot (L.) medicus*.**

1 Rutin, 2 hyperoside, 3 isoquercetin, 4 Myricetin, 5 quercetin-3’-O-glucoside, 6 quercetin


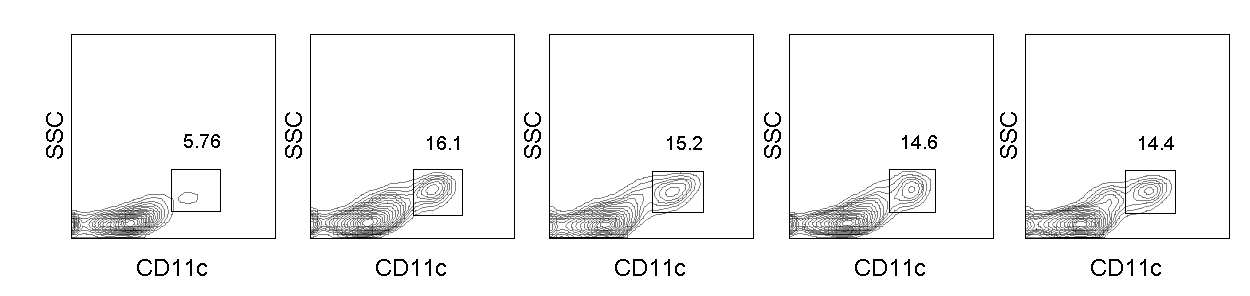
**Supplementary Figure 2**


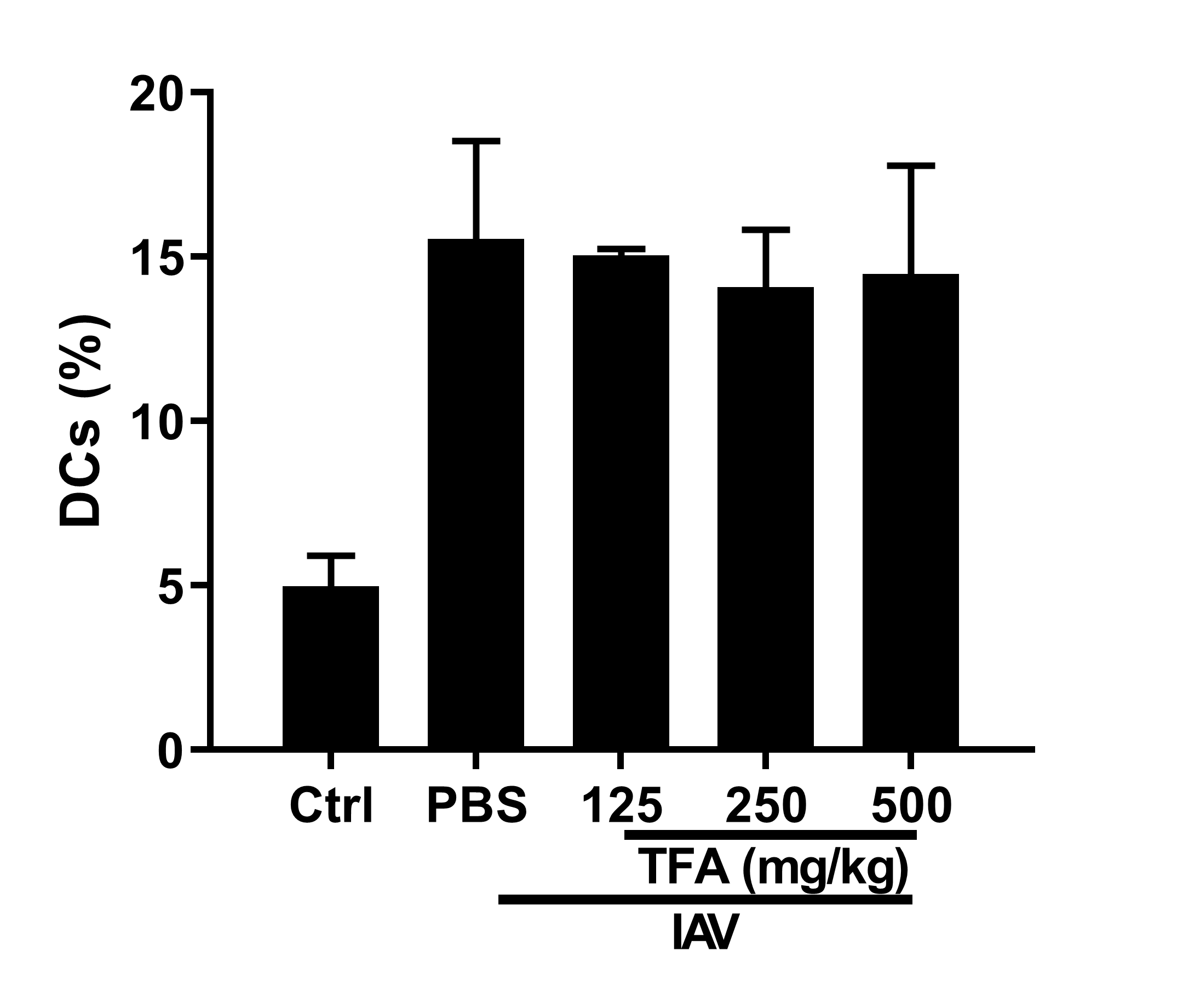


**The proportions of dentric cells in BALF.**

TFA (125, 250, 500 mg/kg) or PBS was gavage daily for 7 days, and PR8 was intranasally administrated at a dose of 5000 TCID50 per mouse (n = 3 mice per group). The proportions of CD11c+ dentric cells in BALF were detected by flow cytometry. The value are shown as means ± SD of three individual experiments.
