# Supplementary material for: Exploring the total flavones of Abelmoschus manihot against IAV-induced lung inflammation by network pharmacology
Source: BMC Complement Med Ther. 2022 Feb 5;22:36. doi: 10.1186/s12906-022-03509-0 (PMC8817495; doi:10.1186/s12906-022-03509-0)

Supplementary Figure 3

Gels/blots are cropped for the clear presentation of results. Samples derived from the same experiment and gels/blots were processed in parallel.

The Uncropped Blot in Figure 5O. The red arrow indicates the location of target bands.

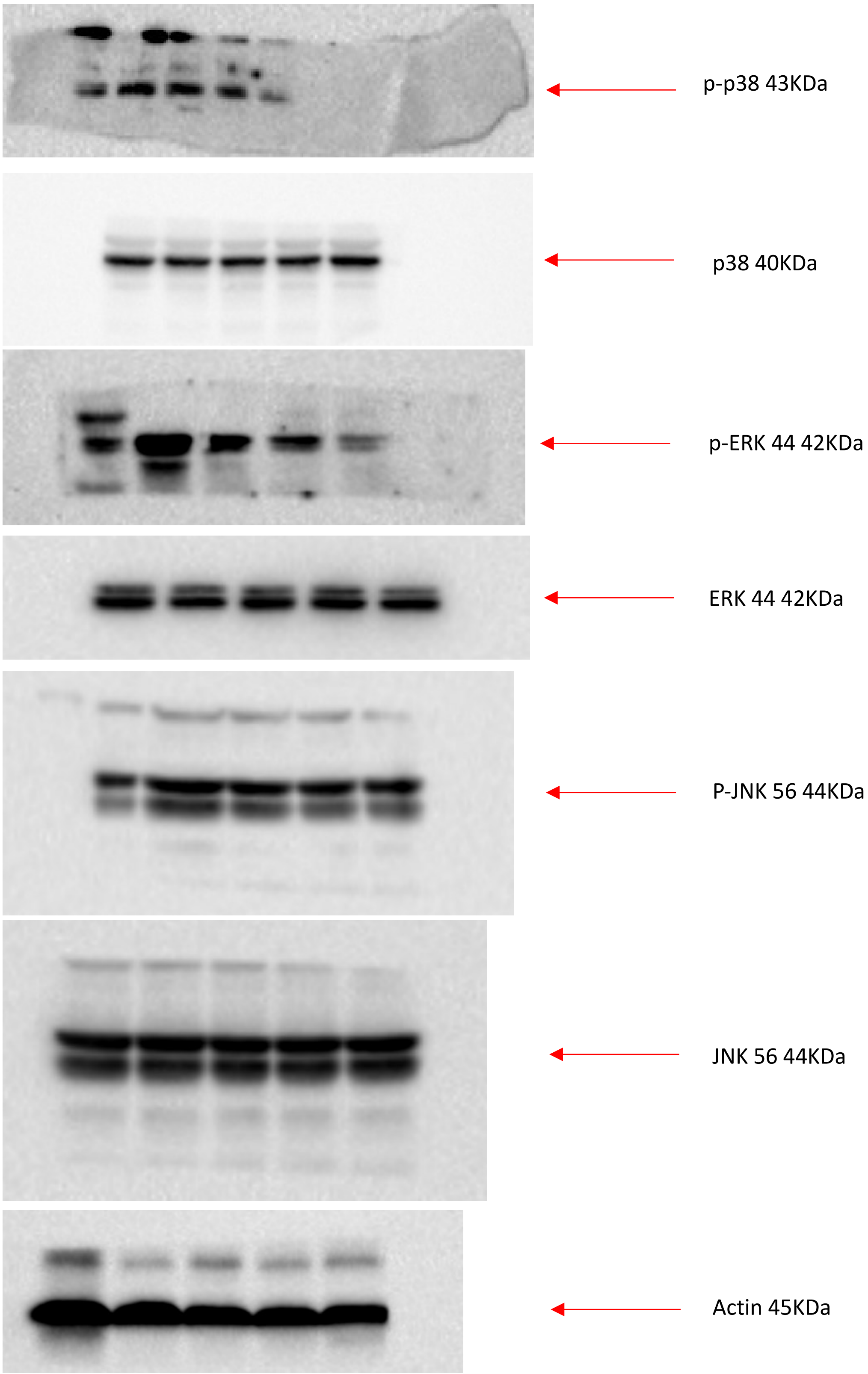

Supplementary Figure 4

Gels/blots are cropped for the clear presentation of results. Samples derived from the same experiment and gels/blots were processed in parallel.

The Uncropped Blot in Figure 6O. The red arrow indicates the location of target bands.

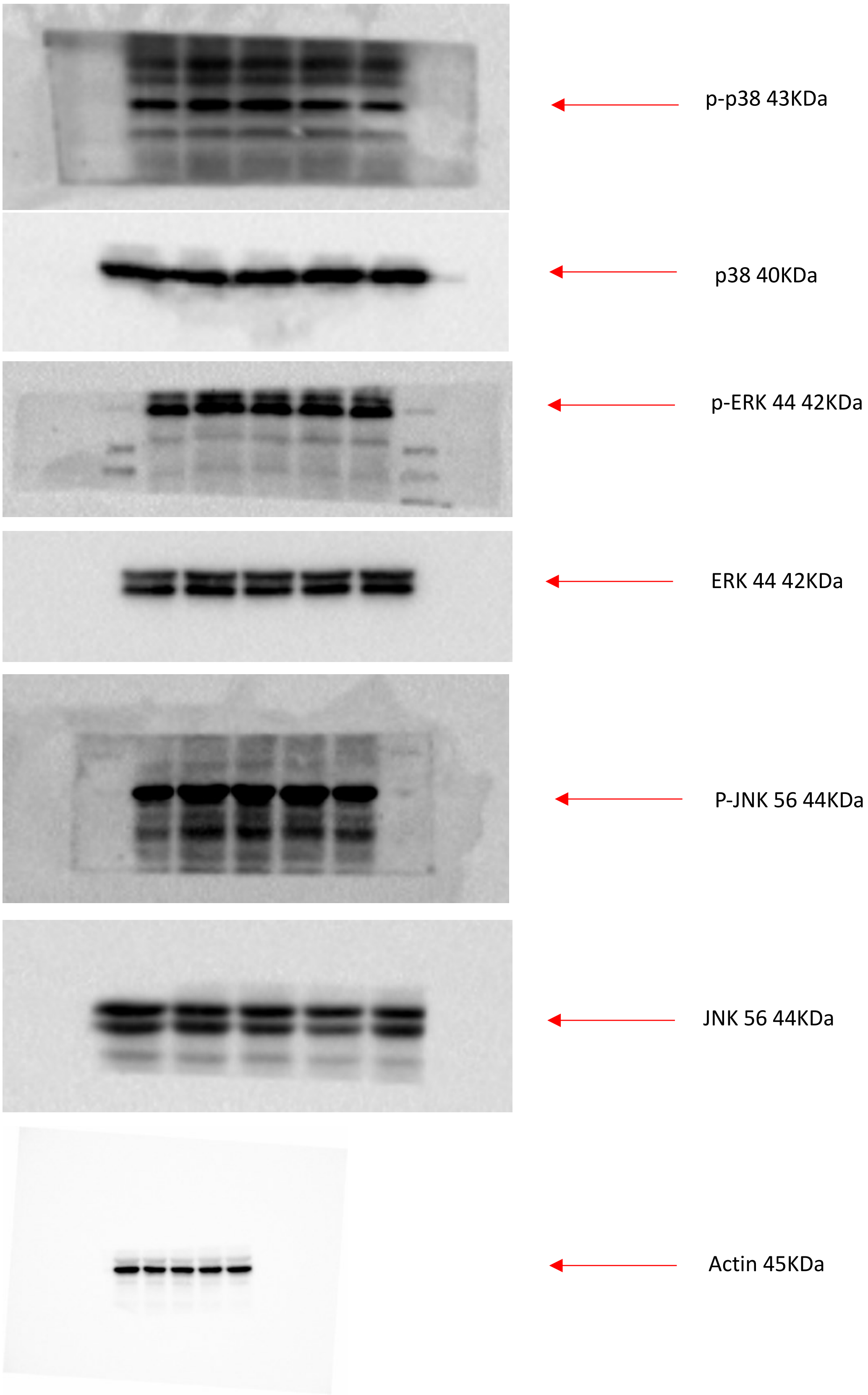

Supplementary Figure 5

Gels/blots are cropped for the clear presentation of results. Samples derived from the same experiment and gels/blots were processed in parallel.

The Uncropped Blot in Figure 7J. The red arrow indicates the location of target bands.

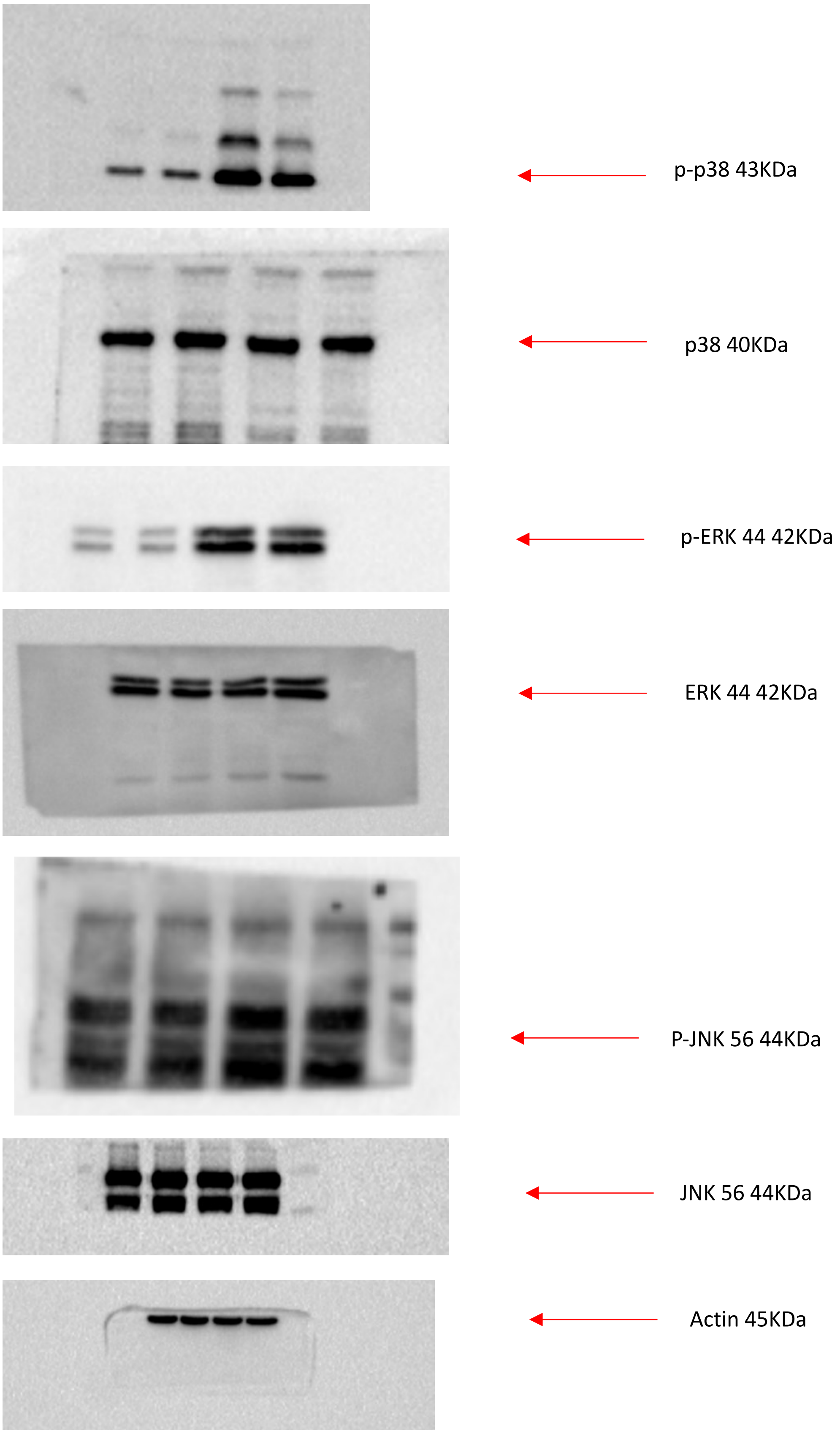

Supplementary Figure 6

Gels/blots are cropped for the clear presentation of results. Samples derived from the same experiment and gels/blots were processed in parallel.

The Uncropped Blot in Figure 7K. The red arrow indicates the location of target bands.

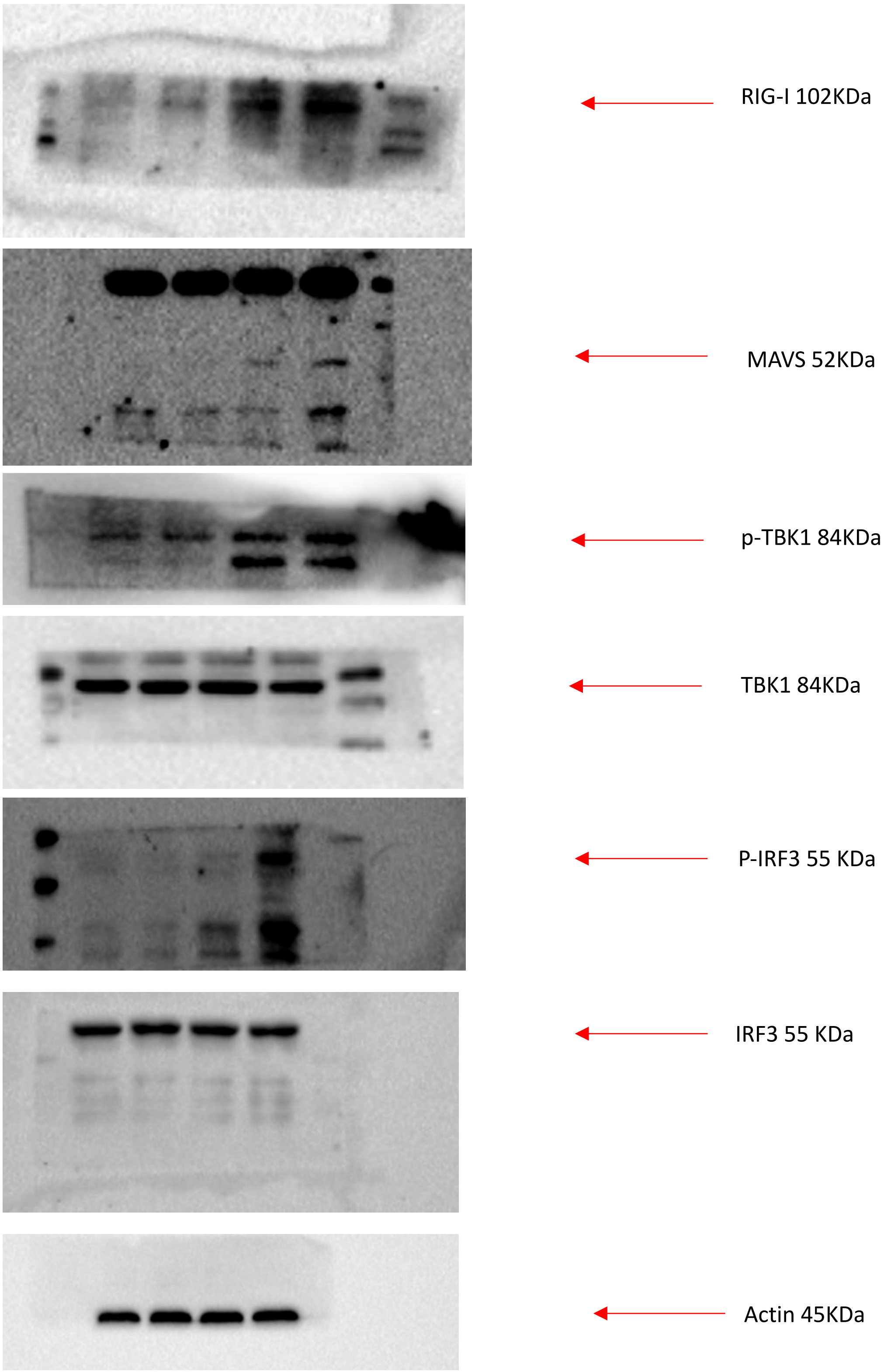

Supplement: Supplementary file 2 — Additional file 2: Supplementary Figure 3. Gels/blots are cropped for the clear presentation of results. Samples derived from the same experiment and gels/blots were processed in parallel. The Uncropped Blot in Fig. 5O. The red arrow indicates the location of target bands. Supplementary Figure 4. Gels/blots are cropped for the clear presentation of results. Samples derived from the same experiment and gels/blots were processed in parallel. The Uncropped Blot in Fig. 6O. The red arrow indicates the location of target bands. Supplementary Figure 5. Gels/blots are cropped for the clear presentation of results. Samples derived from the same experiment and gels/blots were processed in parallel. The Uncropped Blot in Fig. 7J. The red arrow indicates the location of target bands. Supplementary Figure 6. Gels/blots are cropped for the clear presentation of results. Samples derived from the same experiment and gels/blots were processed in parallel. The Uncropped Blot in Fig. 7K. The red arrow indicates the location of target bands. [file 12906_2022_3509_MOESM2_ESM.pdf]
